# What’s Wrong with Gazanias? A Review of the Biology and Management of Weedy Gazania Species

**DOI:** 10.3390/plants14060915

**Published:** 2025-03-14

**Authors:** Babar Shahzad, Muhammad Adnan, Ali Ahsan Bajwa

**Affiliations:** La Trobe Institute for Sustainable Agriculture & Food (LISAF), Department of Ecological, Plant and Animal Sciences, AgriBio, La Trobe University, Melbourne, VIC 3086, Australia; b.shahzad@latrobe.edu.au (B.S.); m.adnan@latrobe.edu.au (M.A.)

**Keywords:** *Gazania linearis*, *Gazania rigens*, garden escapes, environmental weeds, invasive plants, weed biology

## Abstract

*Gazania* is a genus of herbaceous plants from the Asteraceae (daisy) family. Native to southern Africa, several species of this genus have been introduced to different countries as ornamental garden plants due to their beautiful flowers. In the wild, *Gazania* species have been observed with flowers of different shades of pink, red, yellow, orange and combination of these colours. Some species of *Gazania* have escaped the gardens and become highly invasive weeds in their introduced range. Invasive, drought-tolerant and prolific seed-producing *Gazania* plants are found in Australia, New Zealand, Algeria, Egypt, Europe and California. In particular, two perennial species, *Gazania linearis* and *Gazania rigens*, commonly known as gazania, have become a major problem in Australia. They have naturalized and are widespread in a range of environments, such as roadsides, pasture/grassland systems, coastal sand dunes, and natural and managed ecosystems. Their seeds and underground reproductive structures are carried along roadsides by slashers, machinery, wind and water, and spread into native vegetation, pastures, horticultural crops and broadacre agronomic crop production systems. Gazania causes significant environmental, production and economic losses in the infested ecosystems. While limited research has been conducted on their biology and invasion ecology, anecdotal evidence suggests that the ability of gazania plants to produce a large number of seeds form thick, dense populations, and tolerate harsh environments, including drought, heat and sub-optimal soil pH, making them persistent, problematic weed species. In addition, perennial growth habit, high genetic diversity and allelopathic potential have also been suggested to facilitate their invasion success, but no research has been conducted on these aspects. Gazania is very difficult to manage, and currently, there are no effective control options available, including chemical herbicides. The lack of knowledge on their biology, invasion pathways and management is hindering the effective management of gazanias. This review compiles and synthesizes currently available information on the distribution, biology, ecology and management of weedy gazania species, with a particular focus on Australia. We also highlight the key knowledge gaps for future research. We believe this information provides researchers and practitioners with an up-to-date account on the weedy aspects of these popular ornamental plants and will help improve management efforts.

## 1. Introduction

Agricultural productivity and natural landscapes face numerous significant challenges, including the impact of weeds and invasive plant species on crop and food production [1,2]. Among the hundreds of plants that humans have introduced in new regions, there is a small proportion that have become aggressive and dominant invaders in the invaded landscapes [3,4]. These invasive plant species pose a significant pressure on agriculture and the environment due to their ability to suppress ecosystem functions [1,5]. Some of the introduced species are superior in their resource use efficiency and growth against native plants to outcompete native vegetation [6,7]. If not managed properly, these invasive species keep spreading, pose serious threats to native biodiversity and cause major damage to agriculture, environment and the economy. For example, escaped garden plants that have become major invasive weeds are estimated to be costing the Australian economy AUD 4.3 billion annually [8].

One such group of invasive species, *Gazania* spp., has shown some extensive expansion and signs of invasion in southern Australia and beyond, making it a noxious and difficult-to-manage weed [9]. Gazania is known by various common names, including coastal gazania, treasure flower, daisy and tufted gazania [10]. Native to southern Africa, several species of the genus *Gazania* have been introduced globally as ornamental plants [11]. However, these species have escaped from gardens and natural strips into natural and managed ecosystems in their introduced range. In Australia, *Gazania rigens* (L.) Gaertn. and *Gazania linearis* (Thunb.) Druce are the two dominant species of genus *Gazania* and are commonly known as gazania weed or gazanias [12]. These plants are often mistaken as native wildflowers. There are anecdotes that the two weedy species hybridize in the wild as evident from a high genetic variability within the group K–R complex and a wide range of floral colours, including different shades of pink, red, yellow, orange and a combination of these colours. If true, potential hybridization could lead to increased genetic variability, adaptability and invasiveness in weeds such as gazania [13,14].

Originally grown in gardens and natural strips, gazania is widespread and naturalized in a variety of habitats, including coastal sand dunes, roadsides, disturbed sites, pastures, grasslands and natural ecosystems [15]. Due to its obnoxious nature, negative impacts on agriculture and environment, and rapid spread, gazania has gained extensive attention in Australia in recent years [15]. With its increasing environmental and ecological impacts, gazania has been declared as a major environmental weed species in South Australia, while its infestations have been observed in most other states, including Victoria, Tasmania, Western Australia, Queensland and New South Wales [16].

Anecdotal evidence suggests that the successful invasion of gazania in outcompeting native species and establishing itself rapidly in new areas are attributed to its key invasive features such as (1) rapid growth and diverse reproduction mechanism, (2) high seed production and efficient dispersal, (3) adaptability to diverse and disturbed habitats, and (4) tolerance to harsh environmental conditions, e.g., drought, salinity and high temperatures [17,18]. Human-mediated dispersal and a lack of natural enemies have further exacerbated its invasion in the introduced range, especially in Australia. However, the mechanisms involved in the invasion success and lack of effective control of gazania are still unknown.

Different management approaches have been tried to control weedy populations of gazania, but the effective control remains challenging and inadequate [17,18]. Therefore, to develop effective control approaches, it is crucial to understand the biology, invasion ecology and impacts of gazania which may aid in exploiting its weaknesses for long-term management. In this review, we explore the biological and ecological aspects of gazania species as a weed, while also summarizing available literature on its impacts and attempted control efforts. This information will support future research by highlighting key knowledge gaps.

## 2. Taxonomy and Nomenclature

Genus *Gazania*, first described by German botanist Joseph Gaertner and named after famous Italian scholar Theodorus Gaza, is a member of Asteraceae family which comprises 16 species, all native to southern Africa [19] and mostly restricted to the Greater Cape Florist Region. This genus belongs to Arctotideae tribe and subtribe Gorteriinae, which includes seven other genera: *Berkheya*, *Cuspidia*, *Cullumia*, *Didelta*, *Gorteria*, *Heterorhachis* and *Hirpicium* [20,21]. According to Howis et al. [11], DNA sequencing revealed that seven out of sixteen species were monophyletic, while the remaining species were included in a poorly resolved clade called the “*krebsiana-rigens* clade—K-R”. Both invasive species focused on in this review (*G. linearis* and *G. rigens*) belong to the highly complex KR group. Morphological variations within this clade greatly exist across the group due to high genetic variability despite very low sequence of divergence, which supports the recognition of several readily distinguishable species. This has led to suggestions for a comprehensive taxonomic revision of the genus [22]. Even though there is currently no consensus on the species distinction, both weedy species present in Australia, i.e., *G. linearis* and *G. rigens,* appear to co-exist and potentially hybridize. Therefore, for simplicity, we have referred to them as “gazania” throughout this review.

## 3. Habitat and Distribution

### 3.1. Native Range

In its native range, gazania occurs in the subtropical and temperate regions of low altitude to sand alpine meadows in South Africa, Eastern and Western Cape, Namibia, Eswatini, Mozambique, Tanzania and Angola [23]. Gazania vegetations frequently come across roadside habitats, coastal areas, sand dunes, garden strips, and naturalised populations in grasslands [23]. Different local common names include “Botterblomme” in Afrikaans, and “Treasure Flowers” in English. According to the Global Biodiversity Information Facility (GBIF) database, a total of 281 and 955 records of *G. linearis* and *G. rigens* have been reported in South Africa, respectively [24,25].

### 3.2. Invasion History and Introduced Range

**Figure 1:**
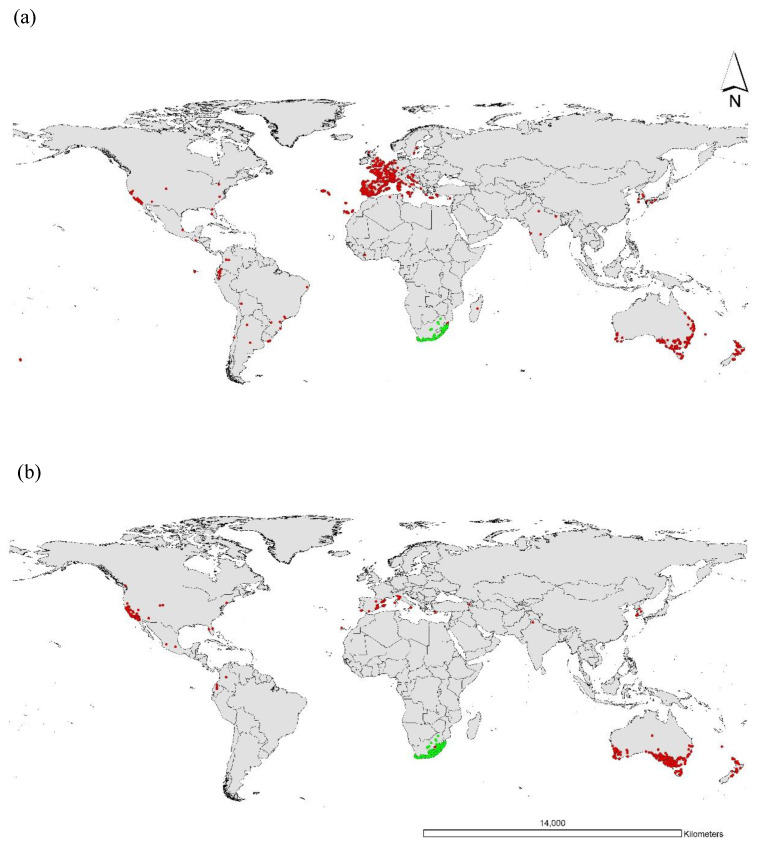
Global distribution of (**a**) *G. rigens* and (**b**) *G. linearis* based on reported occurrence data. Areas coloured red and green indicate the introduced and native ranges, respectively. A total of 3817 occurrences were recorded from 142 datasets, including 910 records from 16 published datasets for native range of *G. rigens.* For *G. linearis*, 2484 occurrences from 120 published datasets, including 1160 records from 20 published datasets, were obtained from the Global Biodiversity Information Facility [24,25].

The early exploration of South African flora resulted in considering several genera of ornamental plants worldwide, including Europe, America and Australia, which have subsequently been used in the trade of horticultural plants globally [11]. Among several other genera, *Gazania* was introduced as an ornamental and ground cover plant due to its spectral and colourful floral display in gardens [26]. Originally cultivated in gardens, it started being established as a weedy plant in locations across its introduced range, including Europe, USA, Australia and New Zealand [9,16] (Figure 1).

In particular, *G. rigens* has shown exceptional invasiveness by spreading across five continents, including Australia, South America, North America, Europe and Atlantic Islands [27]. Major gazania infestations have been reported from California (USA), specifically in counties of San Diego, Los Angeles, Orange and Ventura [28]. A total of 697 records of *G. linearis* have been reported in California [28], indicating a widespread distribution of escaped vegetation, with the potential of additional unreported cases. Although the exact mode of introduction into these areas is unknown, it is believed that many populations of gazania were introduced as ornamentals due to their best-known biological features of tolerance to cold, water scarcity and temperature stress [29]. Today, several populations of gazania are found growing across different habitats such as creekside vegetation and native grasslands [28]. In North Africa, gazania is recognized as an invasive and problematic weed, and has been reported in several countries, including Morocco, Tunisia, Libya and Algeria [30]. Gazania is also reported as an escapee plant with the potential to pose serious threat to agricultural and natural environments in Southern European countries including Madeira, Portugal, Spain and Italy [31,32,33].

Gazania is a widespread weed in Australia and New Zealand, with numerous well-documented infestations [12,16]. *Gazania rigens* and *G. linearis* were introduced in Australia in the 1950s and 1970s, respectively [34]. It is not only a widespread environmental weed, but also an emerging problematic broadacre cropping and horticultural weed in several regions across Southern Australia [17]. The main regions with greater infestations include the Riverland region, York and Eyer Peninsulas in South Australia, central and northern Victoria, and the Malle region of Victoria, South Australia and New South Wales [12,18,35].

### 3.3. Habitat Suitability

The available data and field observations indicate that gazania has the capacity to grow in a wide range of environmental conditions, where it quicky invades and colonises the area [18]. It has been found to grow and flourish across temperate-to-sub-tropical climatic conditions. Gazania has naturalised in a variety of natural and disturbed landscapes, including sandy soils, roadsides, coastal dunes, bushlands, crops and vineyards [18]. Gazania has invaded all Australian states and is particularly well established in South Australia, where it grows as a perennial herbaceous plant with active growth predominantly in spring and flowering in the summer months [15]. Infestation of gazania has been ranked according to their presence in all states. For instance, South Australia has a high ranking for current and future gazania infestations [34]. While field observations suggest that gazanias are adaptable to a wide range of habitats in terms of climatic and edaphic condition, research is lacking on this aspect.

There is no information available on gazania’s growth response to various environmental conditions as a weed. However, the gardening sector has some gazania growing guidelines that indicate suitable growing conditions. For example, it can grow well in many soil types but prefers loose sand and other well drained soil types [36]. Overly moist soil restricts its seed germination [37]. It also prefers soil with a neutral pH (7.0), but it has been observed germinating and growing in alkaline and acidic soils [38]. This explains gazania infestations across sand dunes, coastal cliffs, stream banks and many other disturbed soils [36]. It prefers hot, dry temperatures with a low humidity, and can grow easily in tropical climates [39]. The ideal temperature for its seed germination and growth as garden ornamental has been recommended to be 21–24 °C [37].

### 3.4. Future Projections

The distribution of gazania has been reported widely with potential to expand considerably over the next few decades, especially in Australia. The pattern of expansion and reported occurrences of gazania have shown tremendous increase over the past few years [16,40]. However, certain parts of Australia are more susceptible to gazania invasion than others. For example, *G. rigens* is currently ranked as low-risk with a rating of 14 and 18 for the Australian Capital Territory and the Northern Territory, respectively, while a medium risk rating of 32–36 was allocated to New South Wales, Queensland, Tasmania, Victoria and Western Australia [34]. Of particular concern, *G. linearis* is classified as a high-risk environmental weed in South Australia with a rating of 42 [34]. The classification used in the risk rating is based on the likelihood scale (1–42) of potential establishment and expansion of an invasive species [34]. According to this scale, a rating of ≤24 indicates potential “low risk”, while a rating of 42 is the “high risk” for current as well as future projections of 2035 and 2065 [34]. Despite these modelling predictions, it is unclear how different climate change scenarios will affect gazania invasion in Australia and globally due to lack of empirical data on this particular invasive species.

## 4. Biology

### 4.1. Botanical Description

**Figure 2:**
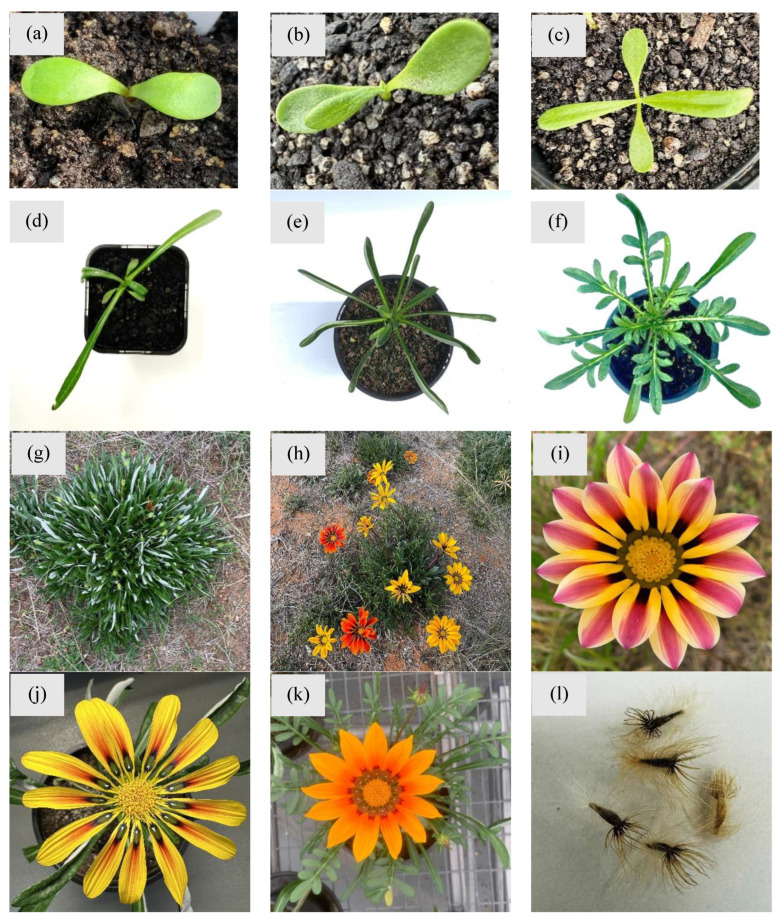
Different growth stages of gazania throughout the typical life cycle. Different growth stages shown above include (**a**) the cotyledon stage; (**b**–**d**) 1, 2 and 5 leaf stages; (**e**) rosette stage; (**f**) active vegetative growth stage; (**g**) fully grown plant with peak vegetative to the early flowering stage, (**h**–**k**) flowering stage with different colour flowers; and (**l**) mature seed production. Photos by Mr. Muhammad Adnan.

Gazania is described as a hairy perennial herb that grows up to 30 cm tall [15]. It is a typical mat-forming plant supported by a main taproot and shallow secondary roots and rhizomes, and spreads as a ground cover. It has upright, oval-to-linear-shaped, waxy leaves that are greenish-hairy and thick, with woolly undersides and succulent with milky fluid. The plant has different growth stages with distinct features, making identification easy (Figure 2).

The plant flowers year-round, but predominantly in spring and summer, producing bright yellow or orange petals occasionally with dark or black bases (Figure 2i,j). While *G. linearis* and *G. rigens* have been reported to present some differences in their morphology and growth habit, it is not easy to distinguish between both species. For example, *G. linearis* appears as a flowering plant with thin linear and slender leaves. The leaves are dark green–silver and very hairy underneath, and measure in 5–10 cm length and 3–20 mm in width [18]. Flowers are typically bright yellow to orange in colour, with ray florets on the underside of the rays. In the case of *G. rigens*, the plant is a creeping perennial herbaceous plant with a milky latex in thick leaves [41]. It bears simple or sparsely branched stems which can grow up to about 50 cm long. Plants produce bright yellow flowers with or without strips or lines, occasionally with dark spots near the base [41]. It is almost impossible to differentiate the species in the wild due to a high level of phenotypic variation and potentially widespread hybridization, meaning these species can readily interbreed.

### 4.2. Life Cycle and Phenology

**Figure 3:**
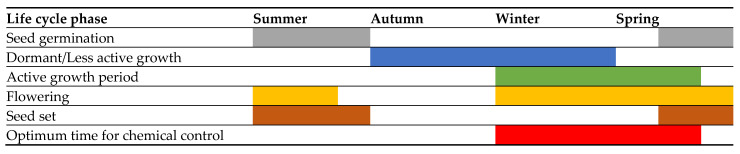
Different life cycle phases or growth stages of gazania across different seasons in the Southern Hemisphere. The shading represents the seasonal growth patterns of gazania [18].

Gazania is a perennial herb that grows year around. The seeds germinate generally in summer after rains, and onset of blooming occurs within a year of germination under favourable conditions [18,36]. Plants usually experience less growth and become dormant in autumn months and vegetative growth begins during and at the end of the winter season (Figure 3). During this period, plants begin establishing their root system and produce important storage reserves that assist in the dormant growth period during the colder months. During the vegetative growth phase, the plants go through a dormant phase while their underground root systems continue to develop [42]. Flowering usually starts from June and spans till December and allows for substantial time for producing small-sized wind-borne seeds [18]. Like most weeds, the onset and timing of various growth stages are often strongly influenced by environmental factors such as temperature and rainfall.

### 4.3. Seed Biology and Dispersal

**Figure 4:**
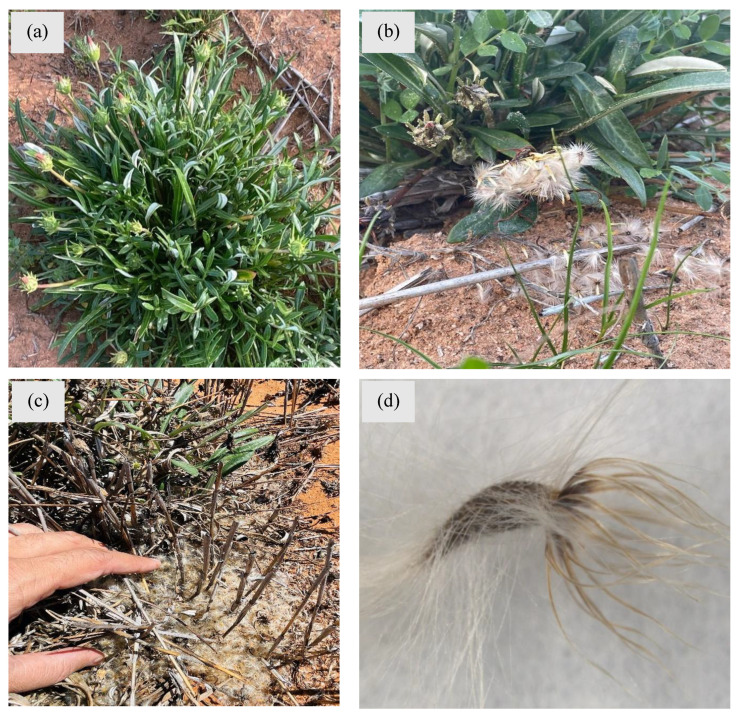
An illustration of the reproductive potential of gazania: (**a**) several flowering buds produced on a single gazania plant, (**b**,**c**) large number of seeds produced by a single gazania plant, and (**d**) microscopic image of a single gazania seed covered with hairs. Photos by Dr. Ali Bajwa (**a**–**c**) and Mr. Muhammad Adnan (**d**).

A single gazania plant can produce 12–15 flowering buds (Figure 4a), but healthy, big plants can produce over 35 flowers under field conditions (*Personal observation*; *Dr. Babar Shahzad*). Each flower can produce up to 60 seeds in one season, indicating the massive seed production ability of this weed [18]. Seeds are generally covered with long hairs that assists in dispersal to long distances by wind, animals, machinery and road grading (Figure 4).

**Figure 5:**
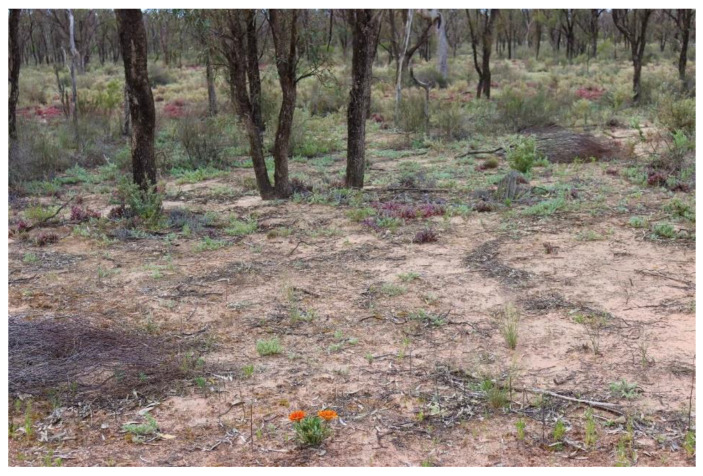
A single gazania plant infests a woodland which is about 2 km away from the nearest gazania infestation. Photo by Dr. Fiona Murdoch, Mallee Conservation.

These wind-borne seeds can spread by wind up to one kilometre away but sometimes seeds drop and germinate near the parent plant, and form a dense ground cover with newly established seedlings [36]. In addition to wind dispersal, other means such as water, animals, vehicles and human clothing also aid in seed dispersal. This way, seeds can be established far from the parent site, which leads to infestations on roadsides and eventually establishes in bushlands, paddocks and grasslands (Figure 5).

Gazania seeds have very little dormancy, but they can undergo dormancy if produced under harsh climatic conditions or have been deposited in areas with unfavourable conditions. In Australia, seeds start to germinate in March and April, before the onset of colder months [36]. If soil temperature and moisture are suitable, most of the seeds tend to germinate within two to three weeks [43]. New seedlings emerge and continue growing underneath the parent plant.

**Figure 6:**
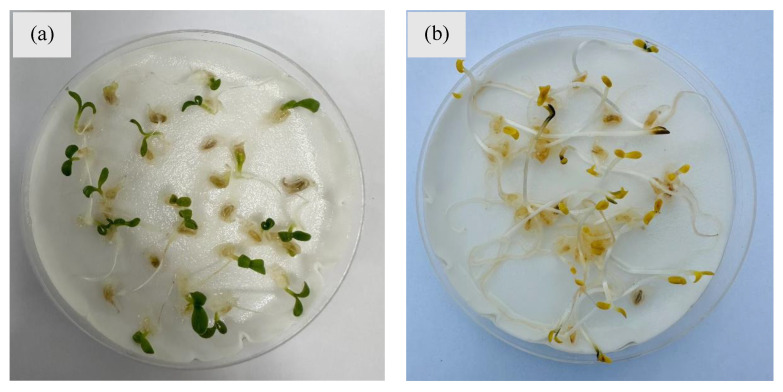
Equally good seed germination of gazania under (**a**) alternative light/dark (14/10 h photoperiod) and (**b**) completely dark (24 h) conditions in the laboratory. Photos by Mr. Muhammad Adnan.

Light is not a necessary component to start its seed germination because gazania seeds start to germinate without light (Figure 6), and sometimes darkness can aid seed germination [44].

**Figure 7:**
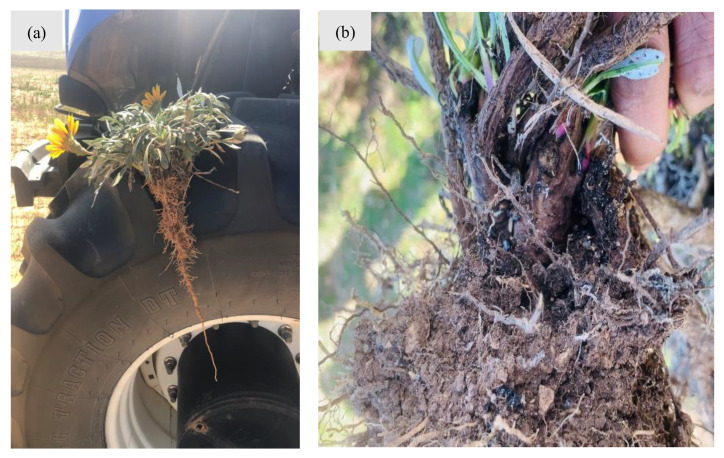
An illustration of the gazania root system: (**a**) long taproot of a relatively small gazania plant at flowering stage and (**b**) extensive secondary roots and some viable rhizomes of a few years-old hardy gazania plant. Photos by Steve Nitschke, a grain grower in the Mallee region, (**a**) and Dr. Ali Bajwa (**b**).

Though gazanias are drought-hardy plants, seeds require an adequate amount of moisture to germinate; however, they cannot tolerate waterlogged conditions at germination and early establishment stages [37]. There is no information available on population dynamics and seedbank persistence, requiring further research on seed biology to better understand the population dynamics of gazania. Gazania has the ability to reproduce by seeds and underground vegetative reproductive structures, commonly called rhizomes [36,45]. Once anchored through its deep taproot, any cuttings or rhizomes can readily establish into healthy new seedlings with the ability to produce flowers and seeds under favourable conditions (Figure 7).

### 4.4. Interference and Stress Tolerance Potential

Gazania possesses several invasive characteristics and has several advantages over other plants, facilitating its quick establishment and significant interference in the introduced areas. For instance, a taproot assisted by a shallow hardy root system not only provides anchorage but also supports the formation of rosette [46]. The rosettes spread horizontally to cover the ground and suppress other vegetation by physically smothering it and by competing for resources [47,48]. Some recently conducted studies have confirmed that gazania plants produce potent secondary metabolites such as steroid-glucoside, alkaloids, polyphenols, terpenes and sesquiterpenes [49,50]. These biochemicals can be toxic to neighbouring plants (allelopathic) depending on the concentration, as documented for other species in the literature [51,52,53]. However, the allelopathic impact of gazania on neighbouring plants remains to be validated through further research.

Gazania plants are extremely hardy and tolerant to many abiotic stresses and exhibit outstanding persistence to withstand and grow in high temperatures, drought, fire, frost, shade and high-salt-stress conditions [36,54,55,56]. Under drought conditions, gazania plants tend to regulate membrane permeability through free proline production and by lowering malondialdehyde contents [54]. Gazania plants also maintain higher water contents through osmotic regulation (stomatal aperture control), and therefore display high water use efficiency under water-limited conditions [41]. Extreme shading conditions are considered unsuitable for gazania; however, it can grow under low light and tolerate moderate shading produced by taller plants growing nearby [36]. Under extreme shade conditions, plants tend to become leggy, with leaves and flowers being closed [55]. It can tolerate salt-laden winds and light frost, but it cannot tolerate severe freezing conditions. Gazania plants were reported to withstand up to 2% of salinity and exhibited minimal growth reduction [56]. Interestingly, salt-treated plants started early flowering (17–20 days compared to control) under 2% salinity [56], indicating the greater adaptability of gazanias under salt stress. In terms of rosette diameter, 1.5–2.9 times lower growth was recorded in saline conditions compared to control plants.

Gazania has the ability to tolerate and become semi-dormant under severe drought conditions, and it quickly resumes growth when there is little moisture available [17]. The ability of this weed to produce thick, waxy, hard, narrow and glossy leaves is probably the most important feature in terms of stress tolerance. These leaf characteristics allow reduced leaf surface area to conserve moisture under extreme temperatures and dry conditions. The difference in the growth patterns of gazania under different environmental conditions may also provide greater advantage over native plants. All these factors and its wide assumed genetic diversity contribute towards its successful interference with natural vegetation, leading to decline in natural biodiversity and successful invasion in new environments.

## 5. Negative Impacts

### 5.1. Environmental

**Figure 8:**
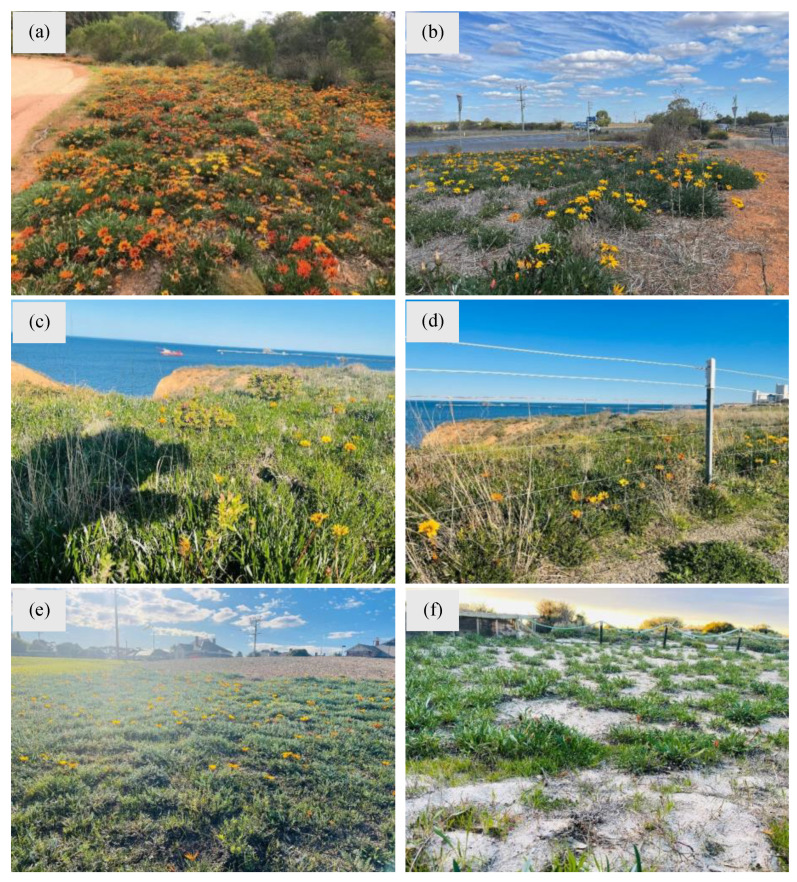
Heavy infestations of gazania across different natural landscapes in Victoria and South Australia. These plants successfully establish (**a**) along roadsides, (**b**) between railway tracks and roadsides in the Mallee region of Victoria, Australia, (**c**,**d**) in coastal areas in South Australia, (**e**) in parks and residential areas, and (**f**) on the beach in Arno Bay, South Australia. Photos by Dr. Fiona Murdoch, Mallee Conservation (**a**) and Dr Ali Bajwa (**b**–**f**).

Gazania has become a problematic environmental weed invading major natural ecosystems and landscapes, as evident from widespread infestations in Australia (Figure 8). Its resilience to readily establish along the roadside verges, coastal areas, sand dunes and disturbed sites dramatically alters the natural balance of areas by outcompeting the native vegetation [11].

It is assumed that vigorous growth, drought hardiness and smothering growth habit aid gazania to outcompete other vegetation by acquiring a major share of key resources such as nutrients, light, moisture and space. Once established in an area, gazania forms dense monocultures largely excluding the native vegetation within short time, which has massive negative impacts on native biodiversity and ecological functions [11]. In Australia, gazania has been recognised among the key threatening processes to high-value native flora across major conservation areas, including the remanent native bushlands, forests, national parks and other natural ecosystems [35]. For example, in parts of north-central Victoria, gazania has infested large stretches of roadsides with flora of high ecological value, including small populations of threatened plant species such as critically endangered spiny riceflower (*Pimelea spinescens* Rye) (*Personal communication: Dr. Ali Bajwa with Ms. Kate Lee*, *Mt Korong Eco-Watch Association Inc. Landcare group*, *Victoria*, *Australia*). These infestations are causing irreparable ecological damage to native ecosystems such as the grey-box forests (*Eucalyptus macrocarpa* Maiden.) preserved by indigenous people of the *Dja Dja Wurrung* tribe centuries ago.

Another environmental impact of gazania is the potential reliance on different types of herbicides to control its expansion in introduced areas. Environmental issues relating to chemical control in natural ecosystems cannot be ignored. Despite the clear field observations and several anecdotes of significant ecological damage being caused by gazania across southern Australia and potentially in other areas, no research has been conducted to assess and quantify these impacts. This has somewhat curtailed the legislative action and prioritisation of this weed for immediate regional and national management.

### 5.2. Agricultural

**Figure 9:**
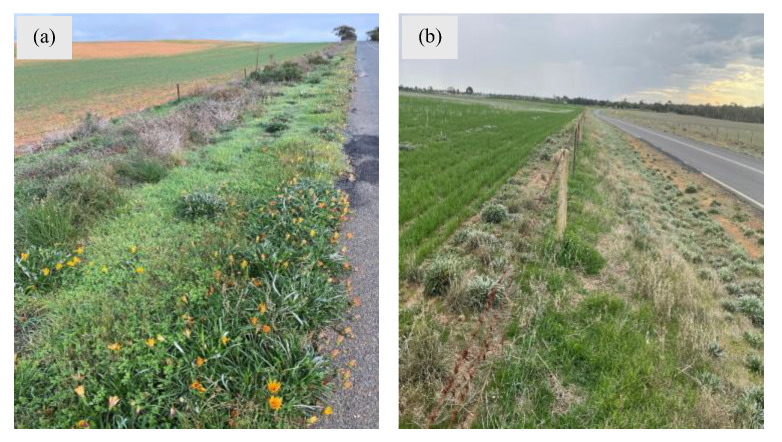
Typical gazania populations growing on the fence lines of grain crop production fields in (**a**) York Peninsula and (**b**) Mallee region of South Australia. Photos by Mr. Chris Davey, Next Level Agronomy (**a**) and Dr. Ali Bajwa (**b**).

Gazania has started to transition from environmental settings to agricultural areas. In Australia, it has infested horticultural tree crops and vineyards for a long time [17], but in recent years, it has started to encroach into broadacre grain production systems [40]. The continuous journey from gardens to the roadsides and coastal dunes to agricultural areas demonstrate that gazania is a highly adaptable and invasive plant which can grows in many soil types and climatic conditions [15]. Due to its exceptional reproductive capacity, vigorous growth, drought tolerance and perennial growth habit, it flourishes and has been expanding in rainfed crop production systems and rangelands across southern Australia [18]. It has been noticed to slowly move from roadsides to firebreaks to fence lines of cropping farms and eventually into cropping fields (*Personal communication: Dr. Ali Bajwa with Mr. Chris Davey*, *Next Level Agronomy*; Figure 9).

**Figure 10:**
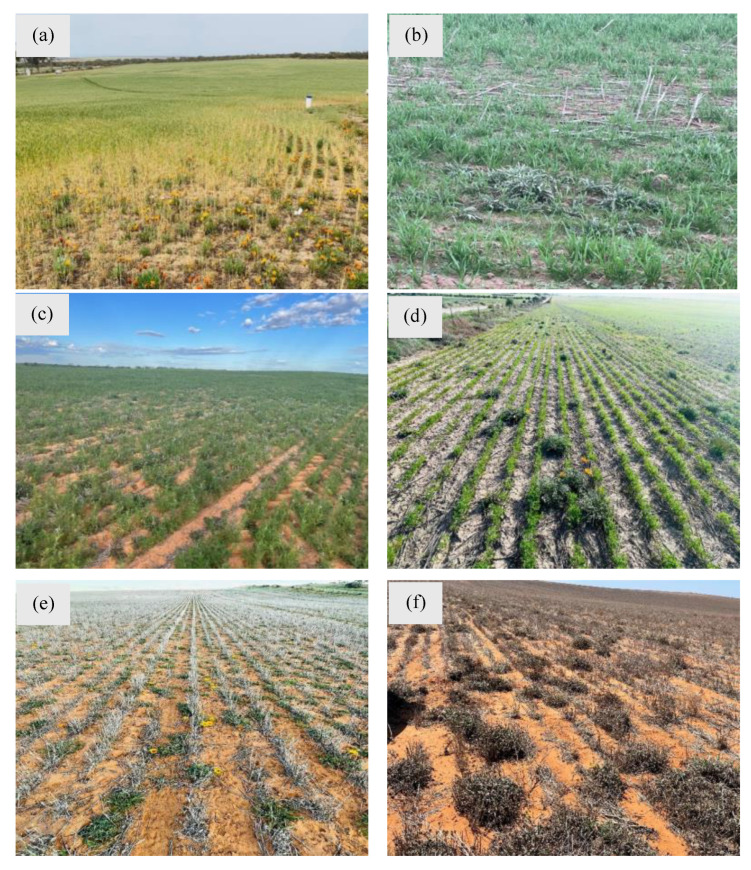
Gazania infestations in (**a**,**b**) wheat (*Triticum aestivum* L.), (**c**) vetch (*Vicia sativa* L.) and (**d**) lentils (*Lens culinaris* L.) crops, (**e**) after crop harvest and (**f**) during the summer fallow phase of the cropping system in different regions of South Australia. Photos by Mr. Sean Mathewson, Dodgshun Medlin (**a**) and Dr. Ali Bajwa (**b**–**f**).

Currently, gazanias can be noticed infesting several major agronomic crops, as well as during the fallow phase in the Mallee region, York Peninsula and Eyre Peninsula in South Australia (Figure 10). Farmers have reported major yield losses and significant expenditure on managing cropping infestations of gazania. However, the full extent of gazania infestations in crop production systems is unknown yet. The quantitative data on actual production losses are also lacking at this stage.

## 6. Management

### 6.1. Biosecurity and Prevention Measures

Proper regulatory framework and policies help prevent the introduction of new invasive plants and weeds. Despite having very strict biosecurity laws, Australia is prone to the introduction and spread of invasive species like gazania. There has been some progress on legislating gazania control in recent years. For example, gazania was recognised as a declared weed under the Landscape Act 2019 of South Australia; therefore, it is illegal to grow, sell and transport these plants, while it is mandatory to control any existing infestations [36]. However, certain varieties of gazania that are claimed to be sterile are allowed to be grown in gardens. It is also rated as a “very high risk” weed in the Advisory List of Environmental Weeds in Victoria [57]; however, gazania seeds and plants are still being sold in nurseries and supermarkets. This makes their management extremely difficult and warrants stronger policy regulations [8].

To control and minimize the spread of invasive weed species such as gazania, it is imperative to quickly identify the newly emerging populations for appropriate control. Modern weed detection systems such as the use of drones, specialised camera imaging and machine learning techniques can be helpful for this purpose [58]. Due to distinctive botanical features, including leaf shape and colour, distinct flowers and year-round growth habit, gazania could be a suitable weed species for the use of modern technology for early detection, mapping and precision weed control. However, no research has been undertaken on these aspects for gazania so far.

To reduce the environmental impacts of gazania and its growth, general biosecurity and best-practice weed prevention measures should be adopted as follows [59]:

1. Regular surveillance activities to monitor and control infestations near hotspot areas;
2. Practice good on-farm biosecurity measures, including the cleaning of farm machinery, careful movement of stock, hay, seed and other materials;
3. Opportunistic monitoring of the sale of gazania in the declared areas and restricting gazania-infested lawn/garden waste;
4. Better management of roadside and fence line populations, especially near agricultural farms;
5. Grow competitive pastures or crops while effectively controlling weeds during the fallow phase;
6. Coordinated awareness and education for public and farmers about the negative impacts of gazania and preventive measures to stop its spread.

While these recommendations are generally true for most weeds, it is important to re-emphasise these points for invasive species like gazania that is widespread in environmental settings while in its early invasion stage for agricultural production areas.

### 6.2. Mechanical Control

The perennial growth habit and the ability of gazania to establish through seeds as well as vegetative reproductive structures like rhizomes make it extremely difficult to control. To restrict reproduction and its further spread, it is advised to mechanically eradicate gazania plants to help controlling the small and isolated populations [18]. Although this method is suitable for smaller, isolated populations, it is not feasible on a large scale. While mechanically removing gazania plants, careful considerations must be given to remove the entire plant including shoot and root systems, as these plants can regenerate/regrow from the rootstocks within the soil [17,18]. The soil surrounding gazania plant(s) should be properly dug to remove the whole plant and be disposed in a way that seeds or rhizomes do not infest new areas. Composting for gazania infested lawn/garden waste can be a viable option. In South Australia, some community level initiatives have been established by the local government authorities to manage gazania in urban settings (e.g., *Gazania Free Gardens* project by Green Adelaide) [60].

It has also been recommended to remove the flowers of gazania plants where possible to avoid seed set [18]. Though manual eradication may seem promising, it is highly labour intensive and may require more resources to cover larger areas [17]. Slashing and mowing have been used to control gazanias in horticultural crops such as almonds (*Prunus amygdalus* Batsch) and grape (*Vitis vinifera* L.) vines with variable success. However, methods such as slashing and cultivation may not be suitable as standalone control methods, as they may spread the plant cuttings and seed-bearing flower heads. Moreover, mechanical removal is also not the best option in no-tillage farming systems.

Mechanical and cultural methods to control gazania only work in case these plants do not widely establish in the field. It is, therefore, highly recommended that uprooted plants must be destroyed before they can re-establish under favourable conditions.

### 6.3. Chemical Control

Chemical herbicides are often employed to control gazania, although control varies under different circumstances [17,42]. In addition, currently no herbicide products are registered for selective control of gazania, especially in agriculture systems. A couple of research experiments have explored various chemical options for controlling gazania in horticulture and fallow situations in Australia, with very few effective control options identified (Table 1).

It has been suggested that gazania should be sprayed when plants are growing actively with lush foliage and flowering to achieve effective control [17]. Chemical applications should be avoided if plants are under any environmental stress such as drought or frost. Under dry conditions, for instance, gazania plants tend to go dormant by closing the stomata and become resistant to herbicide uptake as well as its translocation [17,18]. To achieve adequate herbicide uptake and translocation, herbicides should be applied at an early vegetative growth stage during winter and spring when plants are flourishing due to sufficient rainfall [17,18]. Therefore, it is vital to consider selecting the right time and plant growth stage where possible to achieve the best outcomes in terms of gazania control.

The method of herbicide application is also an important factor which plays an important role in determining herbicide efficacy for gazania control. For instance, herbicide application using high rates of water has been advised to ensure maximum spray penetration in gazanias to achieve their effective control in horticultural crops [17]. In horticultural settings, a tank mix of glyphosate (4 L ha^−1^) and carfentrazone (0.05 L ha^−1^) or higher rates of glyphosate (8 L ha^−1^) provided the effective control of gazania [17]. However, glyphosate application at higher rates was not determined as a cost-effective treatment. Overall, no single treatment provided effective control, and follow-up sprays were advised for maximum control.

Sequential applications (double knocks), for example, the use of glyphosate followed by paraquat/diquat have been shown to cause the severe desiccation of leaves but failed to fully kill the plants and provided them enough time to regrow from the rootstocks [17]. Therefore, it appears that more knockdown options seem inevitable to follow for burning the remaining seedlings. There are currently no reports documented on herbicide resistance in gazania; however, repeated use of the same chemicals should be avoided as a precaution.

In a separate study, complete control (100%) of a roadside population of gazania was achieved by the combined application of 2,4-D (registered as Amine^®^) and chlorsulfuron (registered as Glean^®^) [42]. Despite its effectiveness on young weed seedlings, chlorsulfuron has been nominated for reconsideration by the Australian Pesticides and Veterinary Medicines Authority (APVMA) due to spray drift and potential environmental damage, and longer retention in the soil [61]. Therefore, any use of this herbicide must be considered carefully. Other treatments involving metsulfuron-methyl and isoxaflutole provided up to 80% control, though gazania plants were able to recover from herbicide injury or took long time to show growth suppression [42]. Several other herbicides did not provide good control mainly due to poor chemical interception and translocation. Authors argued that thick waxy leaves of gazania with abundant cuticle layer prevented the uptake and translocation of herbicides. In this regard, further research should focus on the morphology (anatomical adaptations, waxes, cuticle) and physiology of gazania plants for a better understanding of these characteristics to optimise chemical control.

Based on the results of limited studies on the chemical control of gazania and industry feedback, the following points are worth considering while further research can help identify the best chemical control options [17,18,36,42]:

1. Do not spray if plants are suffering from any of the environmental stresses such as drought, frost and heat;
2. Herbicides should be applied at the early seedling/vegetative stage (before plants develop deep root system) or at early flowering stage for maximum control;
3. The use of appropriate surfactants and adjuvants is recommended to increase the absorption and efficacy of herbicides;
4. As single herbicides do not provide effective control, using combinations of herbicides with different mode of actions such as tank mixtures or in sequential applications may provide better control;
5. High volumes of water mixed with herbicides may be used for broadacre applications for better spray coverage, while spot applications with low water volumes and higher herbicide concentrations should be used for smaller/patchy infestations;
6. Apply herbicides at appropriate (optimal) temperature and humidity of air and soil;
7. Only use registered products and strictly adhere to the label requirements.

These are general best practice recommendations based on limited research. It is important to conduct further research on chemical control to validate these principles to achieve effective control of gazania, especially in agricultural systems.

### 6.4. Integrated Management

The use of combinations of all available weed control practices is extremely important for integrated weed management. However, to do that, different chemical and non-chemical (preventive, mechanical and cultural) options must be identified and optimized for the target weed species. Historically, not many control options have been evaluated for gazania, as evident from the above review of the literature. Therefore, not many studies on integrated management are available currently. There has been only one study evaluating 30 different treatments involving combinations of herbicides and mechanical control treatment in vineyards in the Riverland region of South Australia [17]. While no treatment provided full control of gazania, the integrated treatment of mowing before the application of herbicide glyphosate was found to be an effective treatment. However, it was not the most cost-effective option due to additional expenses incurred for mowing gazania [17].

As gazania is transitioning more into agricultural production systems, the need for developing integrated weed management strategies is ever more pressing.

## 7. Conclusions and Recommendations for Future Research, Development and Extension

This review provides insights into the biology, interference, impacts and current management of gazania as an invasive weed species which is becoming a pressing challenge for agriculture and environment in some parts of the world. Clearly, gazania is spreading rapidly across natural and managed ecosystems posing serious threats to native biodiversity, ecological functions, agricultural productivity and sustainability, as evident in southern Australia. A strategic and coordinated management plan is crucial for minimizing the spread and negative impacts of gazania. Currently, there are limited control options available while we also lack information on certain aspects of the biology and invasion ecology of this weed.

Further research, development and extension (RD&E) on the following aspects is recommended to manage this highly invasive plant:

1. Survey and map the existing infestations across natural and agro-ecosystems to quantify the magnitude of the problem and identify hotspots for prioritizing eradication vs. localized control vs. large-scale management. Future studies should also include bioclimatic modelling for identifying the areas where gazania may pose risks in terms of future spread and invasion.
2. Study the seed germination and growth ecology of various gazania populations from different geo-climatic regions and land use situations to understand the life cycle adaptations that are making its continued spread possible as well as complicating its management. A better understanding of biological aspects could help pinpoint important stages to target for management.
3. Invasion and interference pathways and factors enabling them should be studied. For example, determination of competitive traits and potential allelopathic interaction may help quantify true ecological impacts of gazania. It could also reveal potential weak links in plant’s biology which can be exploited for its management.
4. Gazania is still planted as an ornamental plant for its beautiful flowers in many parts of the world and most people are not aware of its invasive traits and significant negative impacts. Therefore, education and extension activities should focus on raising awareness on the issue. Legislative frameworks need to tighten to restrict the trade, movement and growth of species like gazania.
5. Chemical control should be optimised by identifying suitable herbicides, adjuvants and their combinations, especially for gazania control in crop production systems. In addition, research should identify optimal application timing, rates and methods.
6. Integrated weed management strategies should be developed by identifying and integrating potential non-chemical control options such as mechanical/physical control and the use of competitive or suppressive crops and/or native plant species with chemical control. Additionally, biological control should be explored as a long-term option, especially for gazania management in natural ecosystems.

## Figures and Tables

**Table 1:** Chemical options explored for gazania control in Australia.

Herbicide Active Ingredient(s)	Herbicide Group Classification and Site of Action	Control Achieved
Metsulfuron Methyl + 2,4-D Amine + Clopyralid	Group 2 + 4: Acetolactate synthase (ALS) inhibitor + plant cell growth disruptors (auxin mimics)	80%
Imazapyr + Glyphosate	Group 2 + 9: ALS inhibitor + 5-enolpyruvyl shikimate-3 phosphate synthase (EPSPS) inhibitor	30%
Imazethapyr + Glyphosate	Group 2 + 9: ALS inhibitor + EPSPS inhibitor	40%
2,4-D Amine + Chlorsulfuron	Group 4 + 2: Auxin mimic + ALS inhibitor	100%
Halauxifen/Florasulam + Clopyralid + 2,4-D Amine	Group 2 + 4: ALS inhibitor + auxin mimic	80%
Fluroxypyr/Halauxifen + Glyphosate	Group 4 + 9: Auxin mimic + EPSPS inhibitor	50%
Terbutryn + 2,4-D Amine	Group 5 + 4: Photosynthesis inhibition at photosystem II—D1 Serine 264 binders (PS II Serine 264 inhibitor) + auxin mimic	30%
Bixlozone + Glyphosate	Group 4 + 9: Auxin mimic + EPSPS inhibitor	70%
Carfentrazone-Ethyl + Glyphosate	Group 14 + 9: Protoporphyrinogen oxidase inhibitor (PPO inhibitor) + EPSPS inhibitor	High control
Oxyflourfen + Glyphosate	Group 14 + 9: PPO inhibitor + EPSPS inhibitor	Medium control
Saflufenacil + Glyphosate	Group 14 + 9: PPO inhibitor + EPSPS inhibitor	Low control
Paraquat/Diquat + Glyphosate	Group 22 + 9: Photosystem I inhibitor + EPSPS inhibitor	Low control
Isoxaflutole + Glyphosate	Group 27 + 9: Inhibition of 4-hydroxyphenyl-pyruvate dioxygenase (HPPD inhibitor) + EPSPS inhibitor	80%
Bromoxynil/Bicyclopyrone	Group 6/27: Photosynthesis inhibition at photosystem II—D1 Histadine 215 binders (PS II Histadine 215 inhibitor)/HPPD inhibitor	30%
Mesotrione + Glyphosate	HPPD inhibitor + EPSPS inhibitor	30%

Source: Adopted from Bennett [17] and Brougham and Bastian [42]; /sign indicates two active ingredients included in a premix herbicide product; +sign indicates a tank mixture of two different products. Low, medium or high control referred to the ratings provided in the original studies without numerical % control values.
